# Development of a Model for Predicting Mortality Among Patients Hospitalized with COVID-19 During Their Stay in a Clinical Centre

**DOI:** 10.3390/jcm13237300

**Published:** 2024-11-30

**Authors:** Neftalí Guzmán, Pablo Letelier, Camilo Morales, Luis Alarcón, Hugo Delgado, Andrés San Martín, Paola Garcés, Claudia Barahona, Pedro Huenchulao, Felipe Morales, Eduardo Rojas, Dina Guzmán-Oyarzo, Rodrigo Boguen

**Affiliations:** 1 Laboratorio de Investigación en Salud de Precisión, Departamento de Procesos Diagnósticos y Evaluación, Facultad de Ciencias de la Salud, Universidad Católica de Temuco, Manuel Montt 56, Temuco 4780000, Chile; pletelier@uct.cl (P.L.); cbarahona2020@alu.uct.cl (C.B.); phuenchulao2020@alu.uct.cl (P.H.); felipe.morales2020@alu.uct.cl (F.M.); eduardorojasmaturana@gmail.com (E.R.);; 2 Departamento de Procesos Terapéuticos, Facultad de Ciencias de la Salud, Universidad Católica de Temuco, Temuco 4780000, Chile; camilo.morales@uct.cl; 3 Laboratorio Clínico, Hospital Dr. Hernán Henríquez Aravena, Temuco 4780000, Chile; lhuircaleo01@gmail.com (L.A.); h.delgado03@ufromail.cl (H.D.); andres.sanmartin@asur.cl (A.S.M.); 4 Clínica RedSalud Mayor, Temuco 4780000, Chile; garces.paola@gmail.com; 5 Escuela de Tecnología Médica, Facultad de Medicina y Ciencias, Universidad San Sebastián sede Concepción, Concepción 4030000, Chile; dina.guzman@uss.cl

**Keywords:** COVID-19, biomarkers, D-dimer, mortality

## Abstract

**Background:** Various tools have been proposed for predicting mortality among patients hospitalized with COVID-19 to improve clinical decision-making, the predictive capacities of which vary in different populations. The objective of this study was to develop a model for predicting mortality among patients hospitalized with COVID-19 during their time in a clinical centre. **Methods:** This was a retrospective study that included 201 patients hospitalized with COVID-19. Mortality was evaluated with the Kaplan–Meier curve and Cox proportional hazards models. Six models were generated for predicting mortality from laboratory markers and patients’ epidemiological data during their stay in a clinical centre. **Results:** The model that presented the best predictive power used D-dimer adjusted for C-reactive protein (CRP) and oxygen saturation. The sensitivity (Sn) and specificity (Sp) at 15 days were 75% and 71.9%, respectively. At 30 days, Sn was 75% and Sp was 75.4%. **Conclusions:** These results allowed us to establish a model for predicting mortality among patients hospitalized with COVID-19 based on D-dimer laboratory biomarkers adjusted for CRP and oxygen saturation. This mortality predictor will allow patients to be identified who require more continuous monitoring and health care.

## 1. Introduction

The COVID-19 pandemic has been the greatest challenge to public health in recent years. The Americas was one of the worst-affected regions, with a total of approximately 3 million reported deaths, 43.5% of the world total [1]. A recent report by the World Health Organization (WHO) shows that the world life expectancy was reduced by 1.8 years, to 71.4 years, between 2019 and 2021 as a consequence of the pandemic. Healthy life expectancy was also reduced by 1.5 years, to 61.9 years, in 2021 [2].

Although the number of COVID-19 infections and deaths are now tending to decrease, there are still persistent outbreaks, with patients who progress to severe stages or die from the disease as a result of various factors [3]. The appearance of new strains of the virus and insufficient vaccination cover may explain, in part at least, the persistence both of infections and of patients who progress to severe stages of the disease [4].

In response to the need to identify patients at risk of progressing to severe disease or death, several studies have proposed tools for predicting severity and mortality in hospitalized patients [5,6]. Liang 2020 [5], in a study performed in a cohort of 1590 patients, established and validated a clinical score to predict the risk of developing severe COVID-19 at hospital admission, leading to the development of an online tool to optimize medical resource allocation. Although several studies have established prediction models, their generalizability and predictive capacity may be limited as a result of various factors, such as differences between populations [7]. The Chilean population is the result of a mixture of Caucasian, Amerindian, and Negroid populations. Recent studies have shown that the Chilean population has 42% Amerindian ancestry, especially in the northern and southern regions of the country [8]. This evidence justifies the idea of establishing algorithms to support clinical decision-making, based on clinical and laboratory factors, adjusted for a specific population and which can be used by clinical staff as a decision-making tool for treatment and follow-up. 

One of the challenges is to develop simple prediction tools, based on widely used laboratory biomarkers, to allow clinical decisions to be based on accurate patient stratification. Thus, the object of this study was to develop a model for predicting mortality among patients hospitalized with COVID-19 during their stay in a clinical centre.

## 2. Materials and Methods

### 2.1. Study Design and Participants

We performed a retrospective study using a non-probabilistic convenience sample of 201 adult patients (>18 years) hospitalized with diagnosis of COVID-19 at the Dr. Hernán Henríquez Aravena Hospital, Temuco, Chile. The patients were diagnosed according to established criteria, with confirmation by Real-Time Reverse Transcription Polymerase Chain Reaction (qRT-PCR) testing of nasopharyngeal swab samples between March 2020 and April 2021, with follow-up over a total period of 24 months. Exclusion criteria were patients younger than 18 years, pregnant women, and subjects with the diagnosis not confirmed by molecular biology. The study was approved by the Ethics Committee of the Araucanía Sur Health Service (protocol no. 144/2020, July 2020) and performed according to the ethical norms of the Declaration of Helsinki for medical research. The ethics committee authorized the waiver of informed consent.

### 2.2. Data Collection

COVID-19 was defined as a moderate or severe disease on the WHO Clinical Progression Scale [9]. Epidemiological, demographic, and clinical data were obtained from the clinical history of each patient. Demographic variables included the age, sex, origin (urban or rural), oxygen saturation, and respiratory rate, while the clinical history included comorbidities like diabetes, hypertension, obesity, stroke, cardiopathies, etc.

The laboratory results for haematology and haemostasis obtained from the laboratory information system (LIS) were recorded. All the samples for Day 1 were collected within 24 h of admission to hospital. The results were obtained by complete and differential blood count of leukocyte populations using a MINDRAY CAL 6000 haematology analyser (Mindray, Shenzhen, China) to obtain the neutrophil–lymphocyte ratio (NLR) and the platelet–lymphocyte ratio (PLR). The haemostasis tests (prothrombin time, activated partial thromboplastin time, and D-dimer) were performed by coagulation time tests (PT and APTT) and the immunoturbidimetric assay (D-dimer), using a STA^®^ R Max coagulation analyser (Stago, Asnières-sur-Seine, France). Inflammatory biomarkers were measured with the Cobas 8000 modular analyser series (Roche Diagnostics, Mannheim, Germany), by immunoturbidimetric assay (CRP), and electrochemiluminescence (ferritin).

### 2.3. Statistical Analysis

Descriptive statistics were used for the socio-demographic and laboratory characterization of the study population by mortality from COVID-19 (dependent variable). The missing data were handled by multiple data imputation, using the Predictive Mean Matching (PMM) method [10]. Absolute and relative frequencies were used as the qualitative variables. The mean and interquartile range were used as quantitative variables. Inferential analysis of the association of the independent with the dependent qualitative variables was performed using the chi-squared test or Fisher’s exact test as appropriate. The differences of medians according to the variable of interest were measured using the Wilcoxon (or Wilcoxon–Mann–Whitney) test, since the data did not comply with the assumption of normal distribution, corroborated using the Shapiro–Wilk test.

Participant mortality from COVID-19 during their hospital stay was presented by a Kaplan–Meier curve. The differences in the survival curves between the altered D-dimer levels were compared using the log-rank test. Furthermore, a multivariate fit was carried out of the same variable and the D-dimer by epidemiological and clinical variables, besides inflammatory markers using Cox proportional hazards models. Six models were generated, presenting the immediate mortality risk through the hazard ratio (HR) and the 95% confidence intervals. Model 1 was the crude model of the ratio between mortality from COVID-19 and D-dimer. Model 2 was adjusted for CRP, while Model 3 was adjusted for CRP and oxygen saturation. Model 4 was adjusted for CRP, NLR, PLR, ferritin, oxygen saturation, and respiratory rate. Model 5 included, in addition to the variables already mentioned, adjustment for age and sex. Finally, Model 6 extended the previous models by including a history of hypertension, type 2 diabetes mellitus, and stroke as additional variables. These variables have been identified in the literature as covariables related to COVID-19 mortality [11,12]. These models were compared using the Akaike Information Criterion corrected for small samples [13]. 

#### 2.3.1. Model Performance Evaluation

The predictive performance of the selected Cox Model was assessed using several metrics:

(a) Concordance Index (C-index): Evaluated to determine the overall discriminative ability of the model, with values ≥0.7 considered acceptable [14].
(b) Time-Dependent C-Statistic: Assessed the model’s discriminative performance at specific time points (0.125, 0.5, 1, 2, and 3 months) using the method proposed by Uno et al. 2011 [15], accounting for censored data over time.
(c) D-index: Calculated to measure the model’s ability to separate patients who experience the event from those who do not, with values >0.1 indicating acceptable discrimination and >0.3 indicating strong discrimination [16].
(d) Integrated Brier Score (IBS): Evaluated the calibration and overall accuracy of survival predictions over time, where lower values signify better predictive performance. The IBS was compared against a reference model to contextualize the predictive accuracy [17].
(e) Time-Dependent ROC Curve (timeROC): Utilized to observe the predictive quality as a function of follow-up time, complemented by sensitivity (Sn), specificity (Sp), and the area under the curve (AUC) for the specified cut-off points [18].

Internal validation of the Cox Model was performed using bootstrap resampling with 100 bootstrap samples to correct for optimism and assess the stability of the predictive performance metrics. The bootstrap 632+ method was applied when calculating the IBS to obtain an unbiased estimate of the prediction error over time. This approach simulates the process of fitting and evaluating the model on multiple resampled datasets, providing a more accurate assessment of the model’s expected performance [19].

#### 2.3.2. Decision Curve Analysis and Net Benefit

To evaluate the clinical utility of the predictive models, a Decision Curve Analysis (DCA) was performed to calculate the Net Benefit across various probability thresholds. DCA facilitates the comparison of different treatment strategies by considering the trade-offs between true positives and false positives at different threshold probabilities. The Net Benefit was defined as the difference between the proportion of true positives and the proportion of false positives, weighted by the threshold probability [20].

Three treatment strategies were compared: (1) Treat All: Intervention (clinical decision) applied to all patients regardless of predicted risk. (2) Treat None: No intervention applied to any patient. (3) Cox Model: Intervention based on risk prediction from the Cox proportional hazards model incorporating D-dimer, CRP, and oxygen saturation levels.

The analysis was conducted at specific threshold probabilities of 0.05, 0.10, 0.20, 0.30, 0.40, and 0.50 and at distinct time points of 0.125, 0.5, 1, 2, and 3 months. For each combination of threshold and time, the Net Benefit was calculated to assess the clinical advantage of the Cox Model over the reference strategies. The outcomes of the DCA are presented in a summary table and illustrated graphically, providing an overview of the clinical utility of each strategy across varying thresholds and time frames.

Finally, a point risk assessment formula was detailed to generate individualized predictions for each modelled time point, facilitating personalized risk stratification in clinical settings. All statistical analyses were conducted using RStudio software v.4.2.2. A *p*-value of less than 0.05 was considered statistically significant in all comparisons.

## 3. Result

The initial cohort consisted of 201 individuals hospitalized with a COVID-19 diagnosis who were followed to assess the risk of mortality associated with the disease. Table 1 presents the characterization of the study population by mortality from COVID-19. Of the total deaths from COVID-19 (n = 25; 12% of all subjects), 19 were admitted to the Critical Patient Unit (CPU) (18% of admissions), while 6 individuals died in other clinical units of the hospital. On the other hand, in subjects who died late (n = 25), the cause of death was not COVID-19. 

**Figure 1:**
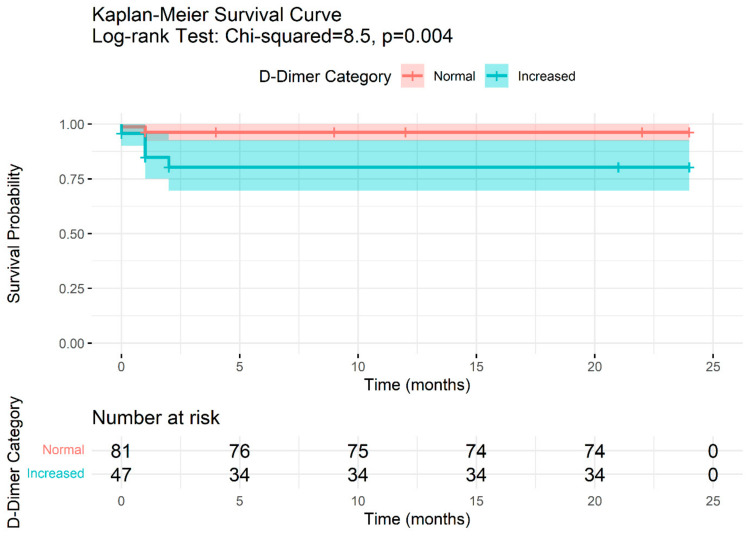
Kaplan–Meier curve of persons with outcome mortality from COVID-19 during the 24 months of follow-up by D-dimer levels.

Participant survival was assessed using Kaplan–Meier curves, stratified by D-dimer levels (Figure 1). In those with normal D-dimer levels, the survival after one month of follow-up fell slightly (98.8%; 95% CI: 96.4–100%), finally reaching 96.3% at the end of follow-up, with a total of two mortality outcomes observed. In the group of participants with elevated D-dimer levels, 47 individuals were observed at the start of the study (time 0 months), with a survival probability of 95.7% (95% CI: 90.1% to 100%). After one month follow-up, the survival dropped to 84.9% (95% CI: 75.1% to 95.9%), with five mortality outcomes observed. After 2 months, the survival probability fell yet further to 80.4% (95% CI: 69.7% to 92.8%), with two further mortality outcomes. The difference in mortality between the two groups was statistically significant (log-rank test *p* = 0.004).

**Figure 2:**
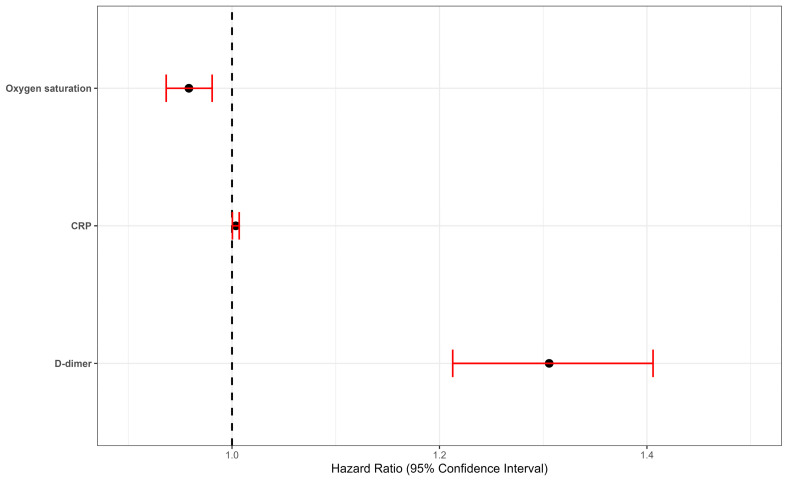
Graphic representation of the best model observed (D-dimer adjusted for CRP and oxygen saturation).

The associations between mortality from COVID-19 and various laboratory biomarkers and epidemiological and clinical variables are shown in Table 2, while the best model observed is represented graphically in Figure 2. Among the six Cox proportional hazards models evaluated, Model 3, which includes D-Dimer, C-reactive protein (CRP), and oxygen saturation, demonstrated the best performance metrics. 

### 3.1. Best Model Performance

The predictive model exhibited robust discriminative performance, as evidenced by a Concordance Index (C-Index) of 0.8180 and a D-Index of 0.1221. These metrics indicate that the model effectively distinguishes between patients at higher risk of mortality and those at lower risk. The calibration and predictive accuracy were evaluated using the Integrated Brier Score (IBS), which yielded a value of 0.085. This low IBS suggests that the model’s survival predictions are accurate over time and demonstrate a lower prediction error compared to the reference model (Model 1; IBS = 0.11). 

Internal validation was performed using bootstrap resampling methods, which corrected for optimism and provided more reliable estimates of the model’s performance on new, unseen data. Specifically, the optimism-corrected C-Index remained at 0.8180, and the D-Index was maintained at 0.12. Additionally, the time-dependent C-statistic showed an incremental improvement over the follow-up period. At early time points of 0.125 and 0.5 months, the time-dependent C-statistic was 0.724, reflecting good discrimination. This value increased to 0.813 at 1 month and further to 0.862 at both 2 and 3 months, indicating excellent discriminative ability in predicting mortality at these later stages, suggesting that the model is particularly effective in forecasting mid-term outcomes in hospitalized COVID-19 patients, thereby supporting its clinical utility for informed decision-making.

Based on the strong performance metrics, a risk assessment formula was developed to estimate the probability of mortality from COVID-19 for individual patients. The established formula is as follows:

*h*(*t*∣*X*) = *h*0(*t*) × (1.31 D-dimer × 1.004 CRP × 0.96 oxygen saturation)

where:

*h*(*t*∣*X*) = hazard function at time *t* for an individual with a set of covariables *X.*

*h*0(*t*) = base hazard function against time for mortality from COVID-19 at time *t.*

Coefficients for D-dimer, C-reactive protein (CRP), and oxygen saturation. Each of these terms indicates how the relative hazard varies with changes in the value of the covariable. 

**Figure 3:**
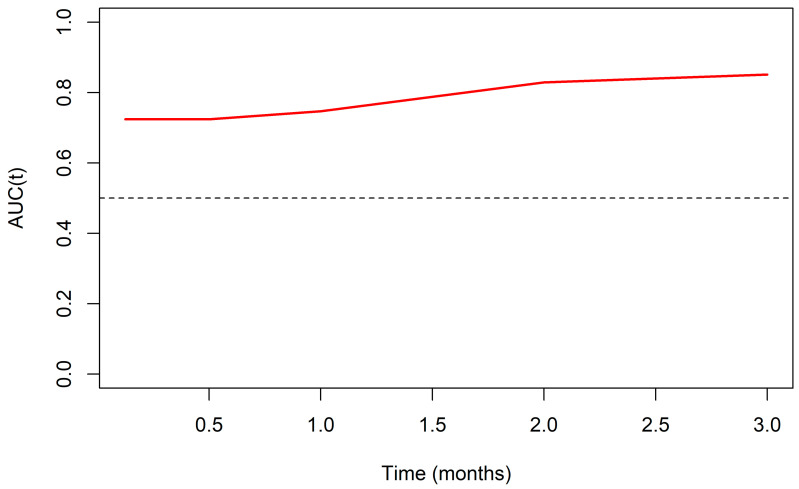
Time ROC curve as a measure of the predictive capacity of the model for predicting mortality from COVID-19 up to 3 months. AUC(t): area under curve at 15 days, 1, 2, and 3 months of follow-up.

Table 3 shows the base hazard values (*h*0(*t*)) at the times when the model is most reliable to facilitate the calculation of the prediction. Finally, Figure 3 shows the time ROC curve for survival or time to outcome data, with points of interest at 0.125, 0.25, 0.5, 1, 2, and 3 months (AUC t = 0.125, 0.25, and 0.5, 72.4%; AUC t = 1.75%). 

The sensitivity and specificity of the model were likewise assessed. Considering the variable with the greatest relative weight, D-dimer, and its cut-off point equivalent to 1.38 μg/mL (previously established for the study population [21]), the Sn and Sp values for t = 0.125 to 0.5 months were 75% and 71.9%, respectively. For t = 1 month, Sn = 75% and Sp = 75.4%. 

### 3.2. Decision Curve Analysis and Net Benefit

**Figure 4:**
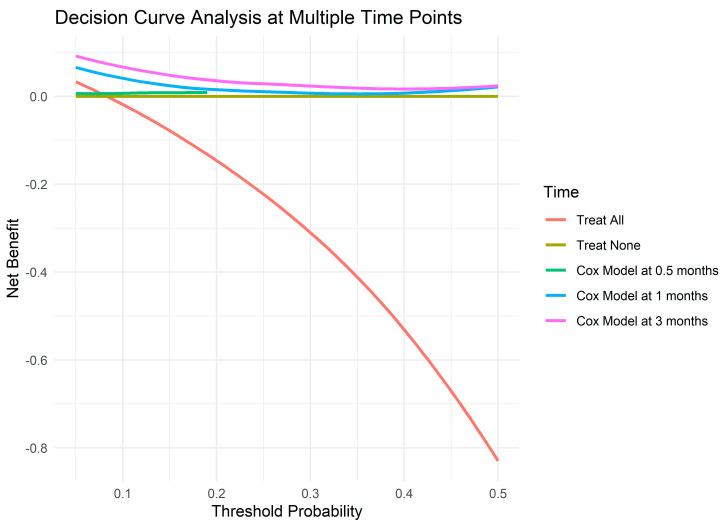
Decision Curve Analysis for treatment strategies across probability thresholds and time points.

To evaluate the clinical utility of the Cox proportional hazards model, a Decision Curve Analysis (DCA) was performed to calculate the Net Benefit across various probability thresholds (0.05, 0.10, 0.20, 0.30, 0.40, and 0.50) and at different time points (0.125, 0.5, 1, 2, and 3 months). The DCA compared three treatment strategies: “Treat All”, “Treat None”, and the Cox Model based on predicted mortality risk. Table 4 presents the Net Benefit values for each strategy at the specified thresholds and time points, while Figure 4 illustrates the Net Benefit curves graphically.

The Decision Curve Analysis (DCA) illustrated in Figure 4 reports that the Cox Model consistently achieves a higher Net Benefit compared to both the “Treat All” and “Treat None” strategies across most probability thresholds and time points. Specifically, at lower thresholds (e.g., 0.05 and 0.10), the Cox Model provides a significant Net Benefit, indicating its effectiveness in identifying patients at higher risk of mortality predicted in the model who would benefit from targeted interventions. As the probability threshold increases, the Net Benefit of the Cox Model remains superior, albeit with diminishing returns, suggesting continued clinical advantages even at higher risk thresholds. 

In contrast, the “Treat All” strategy generally yields a negative Net Benefit at lower thresholds, highlighting potential overtreatment and unnecessary interventions in patients with low predicted risk. At higher thresholds, while “Treat All” begins to show a positive Net Benefit, it does not surpass the performance of the Cox Model. The “Treat None” strategy remains a baseline reference with zero Net Benefit across all thresholds and time points.

It should be noted that, for certain combinations of probability thresholds and early time points (such as 0.125 and 0.5 months), the Net Benefit values for the Cox Model are unavailable, as indicated by “NA” values in Table 4. This is due to the lack of sufficient data or low event rates at these early time points, which limits the estimation of Net Benefit in these specific scenarios. Such cases are omitted from the graphical representation in Figure 4 to provide a clearer view of the available data.

## 4. Discussion

Today, severe cases of COVID-19 are less frequent than in the first years of the pandemic; nevertheless, they continue to constitute a health emergency. Identifying markers for prognosis and developing algorithms based on laboratory parameters is still a challenge. In this study, we developed a model for predicting mortality among patients hospitalized with COVID-19 during their stay in a hospital, based on laboratory biomarkers.

In the population included in this study, no significant differences were observed related with age or sex; this contrasts with other studies, which have shown that age and sex are important predictors of mortality and severe COVID-19 [22,23,24]. Nor were there any significant differences observed by area of origin (urban or rural), which agrees with earlier studies in the Chilean population [25,26].

The results show that the principal biomarkers for statistically significant differences between subjects who did and did not survive are CRP, lactate dehydrogenase (LDH), and D-dimer. Various studies have proposed CRP as a marker of severity and mortality in patients with COVID-19 [27,28]. Stringer et al., 2021 [29], who assessed the CRP response in patients hospitalized with COVID-19, showed that a high level at the moment of admission to hospital constituted an indicator of severe disease, with increased risk of death. Likewise, a study in a Chilean population showed that high CRP at admission to hospital was independently associated with admission to CPU and death (OR = 1.505; 95% CI = 1.107–2.047; *p* = 0.009) [30]. Thus, the potential association between high CRP and mortality would be explained, at least in part, by the presence of hyperinflammation, leading to severe complications like acute respiratory distress syndrome (ARDS), cardiovascular disease, and multiple organ failure [31,32,33]. 

Previous reports have shown the presence of higher LDH levels in patients admitted to CPU or who die [26,30]. It has been shown elsewhere that a high D-dimer level is a predictor of mortality in patients with COVID-19 [34,35]. A retrospective study showed that the highest levels of D-dimer on the day of admission to hospital are associated with worse clinical results, specifically with higher rates of intubation and mortality [36]. A meta-analysis including 66 studies, with a total of 40,614 patients diagnosed with COVID-19, showed that patients with higher D-dimer levels present a worse prognosis than those with lower levels of this biomarker (OR = 4.52; 95% CI 3.61–5.67; *p* < 0.001), making it an independent predictor of mortality from COVID-19 [11]. 

Interestingly, hyperferritinemia is observed in both survivors and in non-survivors, with no significant differences observed between the two groups. A recent meta-analysis evaluated the serum ferritin levels at different levels of severity in COVID-19. When comparing between survivors and non-survivors of COVID-19, a higher level of serum ferritin is observed in non-survivors compared to survivors [37]. However, reports in the Latin American population show contradictory results. In a study carried out in 1418 patients from a Peruvian national reference hospital, no significant differences were observed between survivors and non-survivors, with both groups presenting elevated ferritin levels [38], a result consistent with that observed in this study.

Other models for predicting the progression of COVID-19 have been proposed in the past. In a multi-centre study in China, a score was established (CALL score) based on factors like presence of comorbidity, advanced age (>60 years), LDH, and reduced lymphocyte count (<1.0 × 10^9^ /L), which predicted progression of the disease with good sensitivity and specificity [6]. However, when the CALL score was tested in an Italian population, poor predictive power for severe disease was observed [7]. Recently, a prospective study performed in 592 Chilean patients was used to assess the CALL score; it was found to present good predictive capacity for mortality in the short and long term [39].

It may be observed that the D-dimer shows similar HR and statistical significance values for mortality from COVID-19, after adjusting for different confounders, in all the models presented. In Models 3, 4, 5, and 6, D-dimer, CRP (with a considerably lower risk magnitude and at the border of null effect), and oxygen saturation are the only variables that have a statistically significant association with the result variable. Consequently, for each unit increase in D-dimer level, the risk of mortality from COVID-19 increases by 33%, adjusting for potential confounders (HR 95% CI 1.20–1.47).

If we consider the predictive value of the models established, the most robust model is Model 3, which uses the D-dimer adjusted for CRP and oxygen saturation. This is based on the Akaike Information Criterion corrected for small samples (AICc) and the C-Index. Smaller AICc values indicate a better model, while, in the C-Index, values close to 1 indicate a better predictive capacity. When the time ROC curve for survival data is evaluated, an acceptable AUC is found for the first month of follow-up (AUC t = 0.125, 0.25, and 0.5, 72.4%; AUC t = 1.75%). Adequate sensitivity and specificity values are observed from the D-dimer cut-off point of 1.38 μg/mL established for our population [21]. 

Liang et al. (2020) developed a risk score and web-based calculator to estimate the risk of developing critical illness among patients with COVID-19 based on 10 variables measured on admission to the hospital (AUC in the validation cohort 0.88; 95% CI 0.84–0.93) [5]. Although the AUC is acceptable, it presents the complexity of the variables considered, which include clinical variables, laboratory biomarkers, and chest X-ray. Recently, in a cohort of 673 patients with a median age of 70 years, independent mortality predictors identified in multivariate logistic regression analysis were used to build the 123 COVID SCORE based on three variables: age, oxygen saturation on hospital admission, and percentage of lung involvement in chest computed tomography (AUC 0.774; 95% CI 0.728–0.821; *p* < 0.0001) [40]. Our proposed mortality prediction model presents various advantages: it is based on the use of widely used laboratory biomarkers and clinical data available in clinical centres regardless of their level of complexity, and it allows the risk of mortality to be predicted at the time of admission to hospital, offering support for clinical decisions taken by the health team. However, certain limitations must be considered. First, the study contained a limited number of patients, as it was carried out in a single health centre, and second, the use of a non-probabilistic sample introduced some restrictions on the generalization of the results. The model established could be improved: Validation of this model in a single centre and with a limited sample size could affect the stability of the AUC when applied to larger and more diverse populations. Although the present research provides evidence of the internal validity of the model, it is crucial to perform external validations in multicentre studies or with larger cohorts to ensure its generalization, robustness, and continuous improvement in different clinical scenarios. In addition, vaccination should be considered together with other variables of interest that have emerged as a consequence of progress in knowledge of the disease.

Follow-up of COVID-19 patients using clinical and laboratory parameters combined with predictive models allows the risk to be assessed during the evolution of the disease in order to stratify patients, avoiding the severest forms of the disease; treatment decisions can be anticipated, optimizing health care to reduce patient mortality.

## 5. Conclusions

In conclusion, these results allowed us to establish a model for predicting mortality among patients hospitalized with COVID-19 based on D-dimer laboratory biomarkers adjusted for CRP and oxygen saturation, with an adequate predictive value. This predictive model will help identify patients who should be immediately admitted to the Intensive Care Unit and receive higher levels of care. However, external validation of the model in a larger cohort of patients is necessary.

## Figures and Tables

**Table 1:** Demographic and clinical characteristics and laboratory biomarkers in patients hospitalized for COVID-19.

	Mortality from COVID-19		
Variable	Yes, N = 25	No, N = 176	*p*-Value ^1^
Age, p50. (iqr)	69 (55, 73)	61 (52, 70)	0.110
Sex, n (%)			0.102
Male	8 (32%)	87 (49%)	
Female	17 (68%)	89 (51%)	
Origin, n (%)			0.999
Rural	4 (16%)	31 (18%)	
Urban	21 (84%)	145 (82%)	
Obesity, n (%)			0.601
Yes	10 (40%)	61 (35%)	
No	15 (60%)	115 (65%)	
Cardiovascular disease, n (%)			0.546
Yes	5 (20%)	25 (14%)	
No	20 (80%)	151 (86%)	
High blood pressure, n (%)			0.266
Yes	17 (68%)	99 (56%)	
No	8 (32%)	77 (44%)	
Type 2 diabetes, n (%)			0.304
Yes	11 (44%)	59 (34%)	
No	14 (56%)	117 (66%)	
Admission to Critical Patient Unit, n (%)			0.017
Yes	19 (76%)	89 (51%)	
No	6 (24%)	87 (49%)	
Severity, n (%)			0.001
Moderate	0 (0%)	103 (59%)	
Severe	25 (0%)	73 (41%)	
Oxygen saturation	93 (86, 94)	95 (92, 96)	0.004
Respiratory rate	22 (19, 28)	25 (22, 30)	0.07
WBC (10^9^/L), p50. (iqr)	7.3 (5.8, 10.8)	7.3 (6.0, 9.7)	0.955
NLR, p50. (iqr)	6 (2, 13)	5 (3, 9)	0.859
PLR, p50. (iqr)	169 (108, 361)	201 (135, 316)	0.741
Neutrophils (10^9^/L), p50. (iqr)	6.23 (4.20, 9.33)	5.75 (4.14, 7.78)	0.857
D-Dimer (μg/mL), p50. (iqr)	2.11 (1.39, 5.64)	1.03 (0.66, 1.77)	<0.001
CRP (μg/L)	81 (100)	129 (138)	<0.001
Ferritin (ng/L)	1.000 (1.647)	1.148 (1.353)	0.92
Lactate dehydrogenase (UI/L)	296 (155)	402.5 (136.5)	0.048

^1^ Wilcoxon rank sum test; Pearson’s chi-squared test; Fisher’s exact test. Abbreviations: WBC = white blood cell; NLR = neutrophil–lymphocyte ratio; PLR = platelet–lymphocyte ratio; CRP = C-reactive protein.

**Table 2:** Hazard ratios (CI 95%) for the association between mortality from COVID-19 and the various predictive models calculated from the variables clinical, epidemiological variables, and laboratory biomarkers.

	Model 1			Model 2			Model 3			Model 4			Model 5			Model 6		
Characteristic	HR ^1^	95% CI ^1^	*p*-Value	HR ^1^	95% CI ^1^	*p*-Value	HR ^1^	95% CI ^1^	*p*-Value	HR ^1^	95% CI ^1^	*p*-Value	HR ^1^	95% CI ^1^	*p*-Value	HR ^1^	95% CI ^1^	*p*-Value
D-dimer	1.29	1.21, 1.38	<0.001	1.30	1.21, 1.40	<0.001	1.31	1.21, 1.41	<0.001	1.37	1.24, 1.52	<0.001	1.37	1.23, 1.53	<0.001	1.36	1.23, 1.53	<0.001
CRP				1.00	1.00, 1.01	0.003	1.00	1.00, 1.01	0.003	1.01	1.00, 1.01	0.002	1.01	1.00, 1.01	0.03	1.01	1.00, 1.01	0.03
PLR										1.00	1.00, 1.01	0.4	1.00	1.00, 1.00	0.4	1.00	1.00, 1.01	0.4
NLR										0.97	0.88, 1.08	0.6	0.97	0.87, 1.08	0.6	1.00	0.87, 1.10	0.6
Ferritin										1.00	1.00, 1.00	0.20	1.00	1.00, 1.00	0.2	1.00	1.00, 1.00	0.2
Oxigen saturation							0.96	0.94, 0.98	<0.001	0.95	0.90, 0.99	0.04	0.95	0.90, 0.99	0.04	0.95	0.91, 0.99	0.02
Respiratory rate										1.01	0.97, 1.07	0.64	1.01	0.96, 1.07	0.68	1.02	0.97, 1.08	0.4
Age													1.01	0.97, 1.05	0.7	1.01	0.96, 1.06	0.8
Sex																		
Male													—	—		—	—	
Female													0.81	0.29, 2.24	0.7	0.65	0.23, 1.86	0.4
High Blood Pressure																		
Yes																1.10	0.25; 3.32	0.9
No																—	—	
Diabetes Mellitus type 2																		
Yes																1.70	0.24; 1.43	0.3
No																—	—	
Stroke																		
Yes																3.65	0.02; 4.00	0.4
No																—	—	
AICc	236.10			232.0			229.1			231.9			235.8			239.8		
Concordance Index (standard error)	0.75 (0.05)			0.79 (0.05)			0.83 (0.04)			0.83 (0.05)			0.83 (0.05)			0.83 (0.05)		

^1^ HR = hazard ratio; CI = confidence interval. AICc = Akaike Information Criterion corrected. *p* values < 0.05 were considered statistically significant. Abbreviations: CRP = C-reactive protein; PLR = platelet–lymphocyte ratio; NLR = neutrophil–lymphocyte ratio. Model 1: Crude model of the ratio between mortality from COVID-19 and D-dimer. Model 2: Adjusted in addition for CRP. Model 3: Adjusted in addition for CRP and oxygen saturation. Model 4: Adjusted in addition for CRP, PLR, NLR, ferritin, oxygen saturation, and respiratory rate. Model 5: Adjusted for CRP, PLR, NLR, ferritin, oxygen saturation, respiratory rate, age, and sex. Model 6: Adjusted in addition for CRP, PLR, NLR, ferritin, age, sex, oxygen saturation, respiratory rate, high blood pressure, diabetes mellitus type 2, and stroke history.

**Table 3:** Base hazards for predicting mortality from COVID-19.

Time	*h*0(*t*) *
0.125 (3–4 days)	0.02
0.25 (7–8 days)	0.03
0.5 (15 days)	0.04
1 month	0.07
2 months	0.09
3 months	0.09

* *h*0(*t*) = base hazard function at time of mortality from COVID-19 at time *t*.

**Table 4:** Net Benefit by treatment strategy, probability threshold, and time point.

Probability Threshold	Time (Months)	Treat All	Treat None	Cox Model
0.05	0.125	−0.0264	0	0.0065
0.05	0.5	−0.0264	0	0.0061
0.05	1	0.0537	0	0.0721
0.05	2	0.0755	0	0.0855
0.05	3	0.0755	0	0.0855
0.1	0.125	−0.0835	0	0.0072
0.1	0.5	−0.0835	0	0.0072
0.1	1	−0.0835	0	0.0412
0.1	2	−0.0978	0	0.0334
0.1	3	−0.0978	0	0.0334
0.2	0.125	−0.2189	0	NA
0.2	0.5	−0.2189	0	NA
0.2	1	−0.1237	0	0.0139
0.2	2	−0.1237	0	0.0334
0.2	3	−0.1237	0	0.0334
0.3	0.125	−0.393	0	NA
0.3	0.5	−0.434	0	NA
0.3	1	−0.3028	0	0.0058
0.3	2	−0.3028	0	0.027
0.3	3	−0.3028	0	0.027
0.4	0.125	−0.5728	0	NA
0.4	0.5	−0.5728	0	NA
0.4	1	−0.4302	0	0.0081
0.4	2	−0.4302	0	0.031
0.4	3	−0.4302	0	0.031
0.5	0.125	−0.9502	0	NA
0.5	0.5	−0.9502	0	NA
0.5	1	−0.7979	0	0.0199
0.5	2	−0.7979	0	0.027
0.5	3	−0.7979	0	0.027

NA indicates that no data are available for that specific combination of threshold and time point.

## Data Availability

Data is unavailable due to privacy or ethical restrictions.
